# Impact of COVID-19 on eating habits, physical activity and sleep in Brazilian healthcare professionals

**DOI:** 10.1590/0004-282X-ANP-2020-0482

**Published:** 2021-05-14

**Authors:** Isabella Araújo Mota, Gilberto Diniz de Oliveira, Iuara Paiva Silva Morais, Thamires Ferreira Dantas

**Affiliations:** 1 Universidade Federal da Paraíba João Pessoa PB Brazil Universidade Federal da Paraíba, João Pessoa PB, Brazil.; 2 Centro de Atenção Psicossocial de João Pessoa João Pessoa PB Brazil Centro de Atenção Psicossocial de João Pessoa, João Pessoa PB, Brazil.; 3 Hospital de Emergência e Trauma Senador Humberto Lucena João Pessoa PB Brazil Hospital de Emergência e Trauma Senador Humberto Lucena, João Pessoa PB, Brazil.; 4 Singular Clínica João Pessoa PB Brazil Singular Clínica, João Pessoa PB, Brazil.

**Keywords:** Coronavirus, Eating Habits, Physical Activity, Mental Health, Healthcare Professionals, Novo Coronavírus, Comportamento Alimentar, Atividade Física, Saúde Mental, Profissionais de Saúde

## Abstract

**Background:**

During the coronavirus disease 2019 (COVID-19) pandemic, Brazilian healthcare professionals could have been experiencing impacts on their routine, behavior and mental health.

**Objective:**

To investigate changes in the daily life and sleeping habits of healthcare professionals in Brazil.

**Methods:**

We conducted an observational and cross-sectional study from May to July 2020. A *Google Forms* questionnaire was made available to Brazilian healthcare professionals on the WhatsApp mobile application and through the website of the Brazilian Hospital Services Company.

**Results:**

The sample (n=710) was mostly composed of women (80.8%), aged between 30 and 40 years old (46.6%), predominantly physicians (41.8%) and residing mostly in the state of Paraíba (66.9%), Brazil. Approximately two-thirds of the total sample had some sleep-related complaints, 25.8% due to difficulty initiating sleep, 29.6% due to difficulty staying asleep and 32.5% due to early morning waking. From the population studied, 28.7% (n=204) reported the use of insomnia medication, and 60.3% (n=123) of these were self-medicating. Some participants reported a change in diet (n=557; 78.5%), especially related to the increase in carbohydrate intake (n=174; 24.5%), and 27% (n=192) of the individuals reported an increase of the consumption of alcoholic beverages. Of the total, 561 (81.8%) reported a change in the practice of physical activity.

**Conclusion:**

In this study, Brazilian healthcare professionals showed aspects of quality of life that were more affected during the COVID-19 pandemic than the prevalence seen in surveys of international studies for the general population.

## RESUMO

**Antecedentes:**

Durante a pandemia de COVID-19, os profissionais de saúde brasileiros podem estar sofrendo impactos em sua rotina, comportamento e saúde mental.

**Objetivo:**

Investigar mudanças na vida diária e nos hábitos de sono de profissionais de saúde no Brasil.

**Métodos:**

Estudo observacional e transversal, que ocorreu de maio a julho de 2020. Um questionário da Google (Google forms) foi disponibilizado aos profissionais de saúde brasileiros no aplicativo móvel WhatsApp e no site da Empresa Brasileira de Serviços Hospitalares.

**Resultados:**

A amostra (n=710) foi composta em sua maioria por mulheres (80,8%), com idade entre 30 e 40 anos (46,6%), predominantemente composta de médicos (41,8%) e residentes, em sua maioria no estado da Paraíba (66,9%). Aproximadamente dois terços do total da amostra tinham algumas queixas relacionadas ao sono: 25,8% sobre a dificuldade de iniciar o sono, 29,6% sobre a dificuldade de mantê-lo e 32,5% sobre despertar precoce pela manhã. Da amostra, 28,7% (n=204) referiram fazer uso de medicamentos para insônia e 60,3% (n=123) desses realizaram automedicação. Alguns participantes relataram mudança na dieta alimentar (n=557; 78,5%), principalmente relacionada ao aumento da ingestão de carboidratos (n=174; 24,5%). Observou-se também que 27% (n=192) dos indivíduos relataram aumento no consumo de bebidas alcoólicas. Do total, 561 (81,8%) relataram mudança na prática de atividade física.

**Conclusão:**

Nesta pesquisa, os profissionais de saúde brasileiros apresentaram aspectos da qualidade de vida que foram mais afetados durante a pandemia de COVID-19 do que as prevalências obtidas em pesquisas de estudos internacionais para a população em geral.

## INTRODUCTION

The pneumonia caused by SARS-CoV-2 was first described in Wuhan, a city in southern China^1^, spread nationally and internationally and led to the declaration of pandemic status by the World Health Organization on March 11, 2020, after the registration of more than 118 thousand cases of COVID-19 and 4,292 deaths worldwide^2^. In Brazil, on August 9th, 2020, a level of 100 thousand deaths attributed to COVID-19 was reached, with approximately 1,000 deaths per day since May 19, 2020^3^.

The social isolation used worldwide as an effective prevention from the contagion of COVID-19 has shown several side effects related to mental health^4^, which led to a lack of control of other clinical comorbidities and negative impacts on social, intellectual, cultural, labor and financial aspects^5^. A study carried out in China found that healthcare professionals who were at the fighting forefront of COVID-19 were at high risk of depression, anxiety, insomnia and post-traumatic stress symptoms^6^.

Thus, this study aimed to investigate changes in daily life habits related to sleep, eating and physical activity of healthcare professionals in Brazil to understand the impact of the COVID-19 pandemic on these people’s daily lives. We hypothesized that all healthcare professionals experienced a negative impact on quality of life during the pandemic when evaluating together diet, physical activity, alcohol consumption and sleep. A secondary hypothesis is that physicians would report worse results in sleep habits.

## METHODOLOGY

This was an observational and cross-sectional study that was conducted during the peak of the COVID-19 pandemic in Brazil, from May to July 2020. The research was approved by the ethics committee of the Federal University of Paraiba and by the National Commission for Ethics and Research through Approval No. 4,157,408. A *Google Forms* questionnaire was made available to healthcare professionals from all Brazil through online groups of healthcare professionals on the WhatsApp mobile application and through the website of the Brazilian Hospital Services Company, responsible for managing 41 federal university hospitals^7^. The access to the forms was conditioned to the agreement of the Free and Informed Consent Term, which dealt with anonymity, confidentiality of information, non-mandatory participation in the research and the possibility of quitting the study at any time.

The inclusion criteria were being a health professional and working during the COVID-19 pandemic. There was no exclusion criterion in this study. The electronic form used did not allow filling it out in an incorrect or incomplete way. This study did not receive any specific grant from funding agencies in the public, commercial, or non-profit sectors.

Healthcare professionals answered the Insomnia Severity Index (ISI-7), a questionnaire validated for Brazilian Portuguese^8^ and also validated as an online application tool^9^, in addition to questions about characterization of physical activity and nutritional profile.

### Data analysis

The data were tabulated in a digital spreadsheet and analyzed statistically. Descriptive analysis was performed using measures of absolute frequency, relative frequency, measure of central tendency, mean and standard deviation. Inferential analysis was also performed using the hypothesis test for proportions to obtain the proportion of the frequency of responses to the questionnaire items. The statistical software R, version 3.6.2, was used, and the p-value was determined using Pearson’s chi-square test, considering a statistical significance level of 5% (p<0.05).

## RESULTS

Table 1 contains data referring to the characterization of the 710 respondents, mostly women (n=574; 80.8%), between 30 and 40 years of age (n=331; 46.6%). The survey included healthcare professionals from several backgrounds, mainly physicians (n=297; 41.8%), nurses (n=96; 13.5%), physical therapists (n=79; 11.1%) and nursing technicians (n=73; 10.3%). These professionals were distributed in the federal district and in more than 21 of the 26 Brazilian states and the majority were in the state of Paraíba (n=475; 66.9%).

**Table 1:** Characterization of the sample of healthcare professionals surveyed during the SARS-CoV-2 pandemic.

		n	%
Sex			
	Women	574	80.8
	Men	136	19.2
Age range			
	20–30 years old	159	22.4
	30–40 years old	331	46.6
	40–50 years old	138	19.4
	50–60 years old	59	8.3
	Over 60 years old	23	3.2
Physician		297	41.8
Other healthcare professionals		413	58.2
	Social worker	12	1.7
	Biomedical professional	5	0.7
	Dentist	15	2.1
	Nurse	96	13.5
	Pharmacist	13	1.8
	Physiotherapist	79	11.1
	Speech therapist	39	5.5
	Nutritionist	33	4.6
	Psychologist	21	3.0
	Laboratory technician	1	0.1
	Radiology technician	1	0.1
	Nursing technician	73	10.3
	Occupational therapist	13	1.8
State of professional activity			
	Amazonas	4	0.6
	Bahia	4	0.6
	Ceará	8	1.1
	Distrito Federal	15	2.1
	Espírito Santo	1	0.1
	Goiás	9	1.3
	Maranhão	6	0.8
	Mato Grosso	12	1.7
	Mato Grosso do Sul	2	0.3
	Minas Gerais	10	1.4
	Pará	4	0.6
	Paraíba	475	66.9
	Paraná	25	3.5
	Pernambuco	31	4.4
	Piauí	9	1.3
	Rio de Janeiro	6	0.8
	Rio Grande do Norte	24	3.4
	Rio Grande do Sul	1	0.1
	Roraima	1	0.1
	Santa Catarina	4	0.6
	São Paulo	58	8.2
	Sergipe	1	0.1

The length of professional experience ranged from 1 to 48 years and the work experience at the time of the research included hospital wards for the majority (n=123; 17.3%), followed by intensive care units (n=97; 13.7 %), outpatient clinics (n=92; 13.0%), private clinics (n=90; 12.7%), hospital urgency/emergency (n=62; 8.7%), emergency care units (emergency units) non-hospital emergency services in the public health system in Brazil (n=48; 6.8%), family health units (n=48; 6.8%), surgical units (n=36; 5.1%) and call centers (n=26; 3.7%).

Regarding sleep (ISI-7 questionnaire), most participants reported moderate difficulty in falling asleep (n=215; 30.3%), slight difficulty in maintaining sleep (n=215; 30.3%) and no difficulty with waking up early (n=231; 32.5%) (Table 2). In addition, they showed dissatisfaction with the current sleeping pattern (n=300; 42.3%), with the way this pattern interferes in activities of daily life (n=287; 40.4%), and they also said they were concerned about/stressed with the sleeping problem (n=248; 34.9%) (Table 2).

**Table 2:** Data from Insomnia Severity Index self-reported by healthcare professionals during the SARS-CoV-2 pandemic.

	None		Mild		Moderate		Severe		Very severe	
	n	%	n	%	n	%	n	%	n	%
Difficulty falling asleep	183	25.8	210	29.6	215	30.3	68	9.6	34	4.8
Difficulty staying asleep	210	29.6	215	30.3	195	27.5	68	9.6	22	3.1
Problems waking up too early	231	32.5	176	24.8	186	26.2	78	11.0	39	5.5
	Very satisfied		Satisfied		Moderately satisfied		Dissatisfied		Very dissatisfied	
	n	%	n	%	n	%	n	%	n	%
How satisfied/dissatisfied are you with your current sleep pattern?	42	5.9	190	26.8	87	12.3	300	42.3	91	12.8
	Not at all noticeable		A little		Somewhat		Much		Very much noticeable	
	n	%	n	%	n	%	n	%	n	%
How noticeable to others do you think your sleep problem is in terms of impairing the quality of your life?	55	7.7	137	19.3	163	22.9	287	40.4	68	9.6
	Not at all worried		A little		Somewhat		Much		Very much worried	
	n	%	n	%	n	%	n	%	n	%
How worried/distressed are you about your current sleep problem	88	12.4	104	14.6	371	52.2	113	15.9	34	4.8
	Not at all interfering		A little		Somewhat		Much		Very much interfering	
	n	%	n	%	n	%	n	%	n	%
To what extent does your sleep problem interfere with your daily functioning (e.g., daytime fatigue, mood, ability to function at work/daily chores, concentration, memory, mood, etc.) currently?	69	9.7	112	15.8	205	28.9	248	34.9	76	10.7
Score categories	No clinically significant insomnia		Subthreshold insomnia		Clinical insomnia (moderate severity)		Clinical insomnia (severe)		Total	
	n	%	n	%	n	%	n	%	n	%
	178	25.1	246	34.6	236	33.2	50	7.1	710	100

It was observed that other healthcare professionals had more complaints related to sleep than physicians specifically, although both groups claimed to be dissatisfied with the current sleeping pattern (Table 3), and present moderate difficulty in falling asleep and staying asleep, while most physicians stated mild difficulty or no difficulty. Proportionally, physicians are more dissatisfied with the interference of sleeping problems in their activities of daily life than other healthcare professionals. In contrast, non-medical professionals reported greater dissatisfaction with the perception of others about the interference of sleep with their quality of life and were more stressed with their sleeping problems (Table 3).

**Table 3:** Comparison between the self-reporting of the Insomnia Severity Index between physicians and other healthcare professionals.

Variable		Physicians (n=297)					Other healthcare professionals (n=413)					p-value*
		N	Mi	Mo	S	VS	N	Mi	Mo	S	VS	
Difficulty falling asleep	n	94	99	73	22	9	89	111	142	46	25	0.0001**
	%	31.6%	33.3%	24.6%	7.4%	3.0%	21.5%	26.9%	34.4%	11.1%	6.1%	
Difficulty staying asleep	n	105	100	66	20	6	105	115	129	48	16	0.001**
	%	35.4%	33.7%	22.2%	6.7%	2.0%	25.4%	27.8%	31.2%	11.6%	3.9%	
Problems waking up too early	n	106	73	74	28	16	125	103	112	50	23	0.553
	%	35.7%	24.6%	24.9%	9.4%	5.4%	30.3%	24.9%	27.1%	12.1%	5.6%	
N: none; Mi: mild; Mo: moderate; S: severe; VS: very severe.												
		VS	S	MS	D	VD	VS	S	MS	D	VD	p-value
How satisfied/dissatisfied are you with your current sleep pattern?	n	21	87	30	125	34	21	103	57	175	57	0.282
	%	7.1%	29.3%	10.1%	42.1%	11.4%	5.1%	24.9%	13.8%	42.4%	13.8%	
VS: very satisfied; S: satisfied; MS: moderately satisfied; D: dissatisfied; VD: very dissatisfied.												
		N	L	S	M	V	N	L	S	M	V	p-value
How noticeable to others do you think your sleep problem is in terms of impairing the quality of your life?	n	52	127	21	30	63	100	160	47	25	74	0.029**
	%	17.5%	42.8%	7.1%	10.1%	21.2%	24.2%	38.7%	11.4%	6.1%	17.9%	
How worried/distressed are you about your current sleep problem	n	144	47	9	53	37	212	66	25	35	67	0.003**
	%	48.5%	15.8%	3.0%	17.8%	12.5%	51.3%	16.0%	6.1%	8.5%	16.2%	
N: not at all worried; L: a little; S: somewhat; M: much; V: very much worried.												
		N	L	S	M	V	N	L	S	M	V	p-value
To what extent does your sleep problem interfere with your daily functioning (e.g., daytime fatigue, mood, ability to function at work/daily chores, concentration, memory, mood, etc.) currently?	n	86	102	21	40	39	104	146	55	29	73	0.002**
	%	29.0%	34.3%	7.1%	13.5%	13.1%	25.2%	35.4%	13.3%	7.0%	17.7%	
N: not at all interfering; L: a little; S: somewhat; M: much; V: very much interfering.												
		NI	SuI	MI	SeI		NI	SuI	MI	SeI		p-value
Score categories	n	86	109	86	16	92	137	150	34	0.032*
	%	29%	36.7%	29%	5.3%	22.3%	33.2%	36.3%	8.2%	
NI: no clinically significant insomnia; SuI: subthreshold insomnia; MI: moderate insomnia; SeI: severe insomnia.												

* Pearson’s chi-square test; statistical significance p<0.05;

** statistical significance p<0.05.

When investigating habits and health aspects, 71.3% (n=506) said they did not need medication to adjust their sleep, but 28.7% (n=204) reported using medication, and most of them were self-medicating (n=123; 60.3%) (Table 4). Among the most used drugs were antidepressants (n=54; 7.6%), benzodiazepines (n=44; 6.2%), herbal medicines (n=37; 5.2%), non-benzodiazepine hypnotic (n=28; 3.9%) and melatonin (n=20; 2.8%).

**Table 4:** Data related to habits and general health aspects of healthcare professionals during the SARS-CoV-2 pandemic.

		n	%
Insomnia medication			
	No	506	71.3
	Yes	204	28.7
Medication prescription			
	Total	204	100
	I’m doing self-medication	123	60.3
	There was a medical prescription	81	39.7
Change in diet			
	No	153	21.5
	Yes	557	78.5
Wake up in the middle of the night to eat			
	No	658	92.7
	Yes	52	7.3
Increased consumption of alcoholic beverages			
	No	518	73.0
	Yes	192	27.0
Change in physical activity			
	No	129	18.2
	Yes	581	81.8

Most of the participants stated that they had observed a change in diet (n=557; 78.5%) (Table 4), especially related to the increase in carbohydrate intake alone (n=174; 24.5%), binge eating alone (n=72; 10.1%), and increased nighttime food intake alone (n=37; 5.2%). In addition, many reported, at the same time, an intensification of carbohydrate intake, increasing nighttime food intake and binge eating (n=125; 17.6%). Most respondents stated that they do not wake up in the middle of the night to eat (n=658; 92.7%). Food compulsion, in this study, was not defined as a nosological diagnosis, but as a subjective urgency to eat food in large quantities, followed by a feeling of well-being or relief at first and then by a feeling of guilt.

It was also observed that 27% (n=192) of the individuals reported an increase in the consumption of alcoholic beverages, especially wine (n=121; 14.2) and beer (n=80; 11.2%). Of the total, 561 (81.8%) reported a change in the practice of physical activity, with the majority having stopped exercising (n=383; 53.9%) or reduced the frequency of training (n=183; 25.8 %). Only 9.7% (n=69) of the professionals reported an increase in the frequency of performing physical exercises (Table 4).

## DISCUSSION

The period of data collection for the study, which was from May to July 2020, coincided with the contagion plateau and the number of deaths in Brazil^10^.

A survey conducted in the Brazilian state of São Paulo in 2013, a pre-pandemic period, based on a sample of 1101 adults between 20 and 80 years old, found a prevalence of objective insomnia detected by polysomnography of 32%, a subjective prevalence of insomnia symptoms of 45%, and a subjective prevalence of insomnia detected by Diagnostic and Statistical Manual of Mental Disorders IV (DSM-IV) criteria of 15%. In that study, although women corresponded to 47.3% of the general sample, they also made up 71.4% of individuals with insomnia according to the DSM-IV criteria. That study also found the use of sleep-inducing medication in 5% of subjects with insomnia symptoms, compared to 2% of non-insomnia subjects and 12% of individuals with DSM-IV insomnia. Despite using the ISI-7 scale in its methods, an instrument also used in our work, the cited study did not provide, for comparison of results, the classification of the severity of insomnia or whether the insomnia presented difficulty falling asleep, difficulty staying asleep or problems waking up too early^11^.To our knowledge, there are no surveys about the sleep, eating and physical activity habits, all together, in a reasonable number of healthcare professionals.

An American survey carried out among obese people during social isolation due to the COVID-19 pandemic showed that 47.9% of these people reduced the frequency of physical activity and 55.9% its intensity^12^. Another study collected in Western Asia, North Africa and Europe revealed a 38% reduction in physical activity among people in confinement^13^. In the current study, 53.9% stopped practicing any exercise, while 25.8% reduced frequency or intensity, demonstrating that 79.7% of the healthcare professionals interviewed in Brazil experienced a negative impact regarding the performance of physical activity, a higher level than that for a confined population in other countries during the COVID-19 pandemic up to July 2020^12^^,^^13^^,^^14^.

Ruíz-Roso et al.^15^ reported that physical inactivity in Brazil among adolescents increased from 40.9 to 93% during the COVID-19 pandemic, a finding that is superior to that recorded in Chile, Colombia, Spain and Italy, by the same study.

In addition to being exposed to COVID-19 infection, the sedentary behavior displayed by most Brazilian professionals increases the propensity for morbidity and mortality related to cardiovascular diseases, cancer and an increased incidence of type 2 diabetes mellitus^16^. The fight against physical inactivity through telecommunications, educational materials and government actions has been proposed by scientific entities such as the American Heart Association, the American College of Sports Medicine and the World Health Organization, due to the negative impact on physical inactivity related to the cardiovascular system^17^.

Approximately 70% of the healthcare professionals evaluated in our survey had some complaints related to insomnia, such as difficulty falling asleep, staying asleep or problems to waking up too early. High prevalence rates related to insomnia in the pandemic have also been found in Chinese studies. In the study by Wu^18^, all 60 physicians surveyed, who were COVID-19 frontliners, in their control group, had insomnia with some severity, considering that 61.67% of them corresponded to the score of moderate insomnia. Li, in another study^19^, showed that 58.9% of physicians surveyed in the city of Wuhan and 24.97% in the city of Ningbo showed insomnia with some severity. Lower prevalence of insomnia was reported by Lai et al^6^, whose study reports that 34% of physicians and nurses in their sample had insomnia to some degree, by Zhang^20^, whose study showed a prevalence of insomnia of 36.1% amongst physicians and nurses in Wuhan, and by Wang^21^, whose study found a prevalence of 38% of insomnia amongst physicians and nurses in a pediatric hospital in Wuhan. During the 2002 SARS epidemic, similar prevalence of insomnia among healthcare professionals was observed in Hong Kong^22^ (34.2%) and Taiwan^23^ (37%). Regarding the general population, sleep disorders in the current pandemic were reported by 37.6% of a Greek population sample, with a higher risk for women and residents of urban areas^24^.

Zhang et al^20^ describe the higher prevalence and severity of insomnia in nurses when compared to physicians. In this study, physicians and nurses were, for the most part, dissatisfied with their sleep, and insomnia among physicians was mostly mild, while among nurses, it was moderate, with some difficulty in falling asleep and maintaining sleep. Although physicians have shown greater interference of sleeping problems in their daytime activities, nurses reported greater stress related to these problems. Other studies of previous epidemics found higher levels of stress in nurses than in physicians and a greater probability of increasing the workload in nurses than in physicians^25^^,^^26^. There may be greater chances for physicians to work during the day, so they can sleep well at night, than nurses, whose hospital night shifts may be more frequent^27^. Another study demonstrated that more contact with patients with a more serious disease resulted in IES scores^28^. Physicians generally have less contact with patients than nurses. Nevertheless, it is common in the nursing profession to have more females than males. According to a meta-analysis^29^, women are more susceptible to insomnia, and studies on the current pandemic corroborate this statement^19^^,^^20^. Interestingly, in our study and in most studies found between insomnia and the pandemic of COVID-19^18^^,^^19^^,^^20^, the samples consisted of substantially more women than men, which may have skewed the results.

In our sample, almost 30% of healthcare professionals started using some medication to adjust their sleep during pandemic and more than 50% of these did it through self-medication. A worrying result was the proportion of professionals self-medicating with drugs that would require medical prescriptions to be purchased and are, therefore, being used without any professional monitoring.

Regarding eating habits, the negative impact on Brazilian healthcare professionals seems to be greater than that found in other countries. Almandoz et al.^12^ describe that 12.1% of obese people did not eat any of the daily routine meals during the pandemic, a percentage which is at least 50% lower compared to Brazilian healthcare professionals of our survey. In this study, about half of the Americans interviewed stored food and 61.2% reported stress eating. In Italy, despite almost 50% of respondents reporting increased weight, there was greater adherence to the Mediterranean diet^14^, differently from our study, which showed a 60.8% increase in carbohydrate intake and binge eating in 30.3% of the sample.

The increase in the intake of ultra-processed foods during the lockdown was detected in adolescents living in both Latin America and the European continent^15^. China, on the other hand, showed an increase in the ingestion of vegetables, water, tea, coffee, fruits and grains, as well as a reduction in the consumption of sweet drinks and snacks^4^, due to the impact of campaigns related to food security and nutrition. While Ammar et al. reported a drastic reduction in alcohol consumption among their interviewers^13^, there was an increase in alcohol intake in 27% of Brazilian healthcare professionals in our survey.

The interference of the COVID-19 pandemic with quality of life appears to differ in each country^12^^,^^13^^,^^14^. In Brazil, according to our research, the healthcare professionals studied stopped or reduced the practice of physical activity and increased carbohydrate intake, which increases the propensity for weight gain^30^ and immune compromise^31^^,^^32^. About 40% showed dissatisfaction with the quality of sleep, and among those who started taking medication for sleep adjustment, most of them did it without medical advice.

Healthcare professionals are more susceptible to COVID-19 infection worldwide^33^. On August 14, 2020, the lethality rate in the general population of COVID-19 in the world was 3.64 according to WHO data^2^ and, in Brazil, 3.3% according to the Ministry of Health^3^. Diet, sleep and physical activity, aspects of healthcare professionals evaluated in this study, are involved in the maintenance of body homeostasis and can certainly^34^^,^^35^^,^^36^^,^^37^ act, depending on the adjustment, as a risk or a protective factor for morbidity and mortality and possibly for mortality directly related to the COVID-19 infection.

The analysis of the data did not show significant differences between the results found in the sample from the state of Paraíba with the sample from the other Brazilian states. Despite this similarity of results, the low proportion in the sample coming from the other Brazilian states — 33.1% of the total population studied — when compared to the proportion of the sample coming from the state of Paraíba — 66.9% of the total population studied — indicates a limitation of this study. Additional studies with more individuals from other states than Paraíba are necessary to verify the results found in our study for all of Brazil.

The main limitation of this study was the fact that the questionnaires are self-managed, which can bias the correct data. However, this was the possible research instrument during the period of need for social isolation so that biosafety could be respected. This is a strategy used by much of the current research on COVID-19 that we have consulted in our literature review. In addition, although this research is a cross-sectional study, the lack of a proper pre-pandemic control group is a limitation, and this would contribute to gauging a possible causality between the pandemic and the changes in sleep and habits found in the result. Further limitations are the overrepresentation of females and the relatively low number of participants in some regions.

In this study, Brazilian healthcare professionals displayed aspects of quality of life (physical activity, diet, alcohol intake and sleep) that were more affected during the COVID-19 pandemic compared to the findings of surveys of international studies for the general population. These are worrisome findings that indicate a need for greater assistance intervention for this group of professionals, who are so important to the population, especially in this period of coping with the pandemic.
